# 22q11.2 Deletion Syndrome as a Human Model for Idiopathic Scoliosis

**DOI:** 10.3390/jcm10214823

**Published:** 2021-10-20

**Authors:** Steven de Reuver, Jelle F. Homans, Tom P. C. Schlösser, Michiel L. Houben, Vincent F. X. Deeney, Terrence B. Crowley, Ralf Stücker, Saba Pasha, Moyo C. Kruyt, Donna M. McDonald-McGinn, René M. Castelein

**Affiliations:** 1 Department of Orthopaedic Surgery, University Medical Center Utrecht, 3584CX Utrecht, The Netherlands; s.dereuver-4@umcutrecht.nl (S.d.R.); J.F.Homans-3@umcutrecht.nl (J.F.H.); t.p.c.schlosser@umcutrecht.nl (T.P.C.S.); m.c.kruyt@umcutrecht.nl (M.C.K.); 2 Department of Pediatrics, Wilhelmina Children’s Hospital, University Medical Center Utrecht, 3584CX Utrecht, The Netherlands; M.L.Houben@umcutrecht.nl; 3 Department of Orthopaedic Surgery, The Children’s Hospital of Philadelphia (CHOP), Philadelphia, PA 19104, USA; Vincent.deeney@chp.edu (V.F.X.D.); PashaS@email.chop.edu (S.P.); 4 The Perelman School of Medicine, University of Pennsylvania, Philadelphia, PA 19104, USA; MCGINN@chop.edu; 5 Division of Human Genetics and 22q and You Center, The Children’s Hospital of Philadelphia (CHOP), Philadelphia, PA 19104, USA; CrowleyT@email.chop.edu; 6 Department of Pediatric Orthopedics, Altona Children‘s Hospital, 22763 Hamburg, Germany; ralf.stuecker@kinderkrankenhaus.net; 7 Department of Orthopedics, University Medical Center Hamburg-Eppendorf (UKE), 22763 Hamburg, Germany

**Keywords:** idiopathic scoliosis, 22q11.2 deletion syndrome, human model, neuromuscular scoliosis, radiography, MRI, curve morphology, intraspinal anomaly

## Abstract

To better understand the etiology of idiopathic scoliosis, prospective research into the pre-scoliotic state is required, but this research is practically impossible to carry out in the general population. The use of ‘models’, such as idiopathic-like scoliosis established in genetically modified animals, may elucidate certain elements, but their translatability to the human situation is questionable. The 22q11.2 deletion syndrome (22q11.2DS), with a 20-fold increased risk of developing scoliosis, may be a valuable and more relevant alternative and serve as a human ‘model’ for idiopathic scoliosis. This multicenter study investigates the morphology, dynamic behavior, and presence of intraspinal anomalies in patients with 22q11.2DS and scoliosis compared to idiopathic scoliosis. Scoliosis patients with 22q11.2DS and spinal radiography (*n* = 185) or MRI (*n* = 38) were included (mean age 11.6 ± 4.2; median Cobb angle 16°) and compared to idiopathic scoliosis patients from recent literature. Radiographic analysis revealed that 98.4% of 22q11.2DS patients with scoliosis had a curve morphology following predefined criteria for idiopathic curves: eight or fewer vertebrae, an S-shape and no inclusion of the lowest lumbar vertebrae. Furthermore, curve progression was present in 54.2%, with a mean progression rate of 2.5°/year, similar to reports on idiopathic scoliosis with 49% and 2.2–9.6°/year. The prevalence of intraspinal anomalies on MRI was 10.5% in 22q11.2DS, which is also comparable to 11.4% reported for idiopathic scoliosis. This indicates that 22q11.2DS may be a good model for prospective studies to better understand the etiology of idiopathic scoliosis.

## 1. Introduction

Scoliosis is a deformity of the spine and trunk that can have a clear cause, such as neuromuscular disease or congenital spinal malformation; however, the majority of cases are referred to as ‘idiopathic’ and occur in otherwise healthy adolescents [1]. Idiopathic scoliosis is quite common, with a prevalence of 2–4% in the general population, but its exact etiology remains clouded, despite important recent discoveries about genetics and the role of human upright spinal biomechanics [1,2,3,4,5,6]. Knowing the exact cause(s) is of utmost importance for potential scoliosis prevention and optimal treatment. The problem with current human etiology research is that, by necessity, only patients with an already established idiopathic scoliosis are studied; therefore, it is impossible to distinguish cause from effect [1,3].

Prospective cohort research, which follows the development of scoliosis starting in the pre-scoliotic spine, is practically impossible in the general population due to practical and ethical obstacles: the prevalence of idiopathic scoliosis would require thousands of children to be included for sufficient statistical power, and there would have to be periodic follow-ups with full spine radiographs, raising ionizing radiation concerns. The next best option is to use a ‘model’ with better availability or a higher idiopathic scoliosis prevalence—for instance, an animal model. Unfortunately, idiopathic scoliosis is a disease unique to humans, mainly due to our unique upright spinal biomechanics [7]. Earlier studies demonstrated that the computation of spinal biomechanics, for instance, with finite element models, can help understand idiopathic scoliosis [8]. Additionally, idiopathic-like scoliosis can be established in genetically modified animals such as zebrafish, pinealectomized chickens, or bipedal-forced mice; however, the translatability of this model to the human situation is questionable [9,10,11,12].

To prospectively study the etiology of idiopathic scoliosis, a human model is therefore preferred but has not yet been described. In other fields of medicine, such as psychiatry, the innovative use of a subset of the population with a high risk of a certain disease has been used and validated to serve as a ‘model’ for the disease in the general population [13]. This approach obviously also has scientific limitations, but if the model sufficiently resembles the condition in the general population, it can yield important information on specific aspects of the earliest phases of the disorder that cannot otherwise be studied prospectively.

The 22q11.2 deletion syndrome (22q11.2DS), the most common cause of DiGeorge syndrome, is the most common microdeletion syndrome in humans, with an incidence of 1 in 992 unselected pregnancies and 1 in 2148 live births [14,15,16]. Compared to the general population, these children have a 20-fold increased risk of developing scoliosis during their growing years, with a prevalence of around 50% [17]. Children with 22q11.2DS are often identified before or shortly after birth, well before potential scoliosis onset, are usually known in the pediatric circuit, and could therefore be studied prospectively [18,19]. It is currently unknown if scoliosis in 22q11.2DS sufficiently resembles idiopathic scoliosis in the general population, which is a prerequisite to be used as a ‘model’. This study focused on the morphology, dynamic behavior, and presence of intraspinal anomalies, all of which are quantifiable features relevant to idiopathic scoliosis development. These were studied in 22q11.2DS and scoliosis patient cohorts from multiple centers and compared to what is reported in the literature for idiopathic scoliosis in the general population.

## 2. Materials and Methods

### 2.1. Study Population

The local Ethical Review Boards of the three hospitals involved approved this study and waived the necessity of explicit (parental) informed consent since data were collected as part of standard care and were handled anonymously. In all participating centers, spinal radiographs are made of each patient at two-year intervals as part of a global standard 22q11.2DS follow-up protocol [20].

From databases of two specialized 22q11.2DS centers, patients with an available full spine radiograph were extracted. All patients that were ambulant, had a genetically confirmed 22q11.2 deletion (via FISH, 22q11.2 specific MLPA, CGH, or SNP micro-array), were aged >4, and had scoliosis defined as a Cobb angle >10° were included [21]. Patients with congenital spinal anomalies (based on spinal radiography review) that induced congenital scoliosis were excluded since the pathoetiology varies greatly from the development of idiopathic scoliosis [22]. Additionally, non-ambulant patients (based on the patient’s chart review) were excluded. Sex, age at the time of radiography, and data on comorbidities were collected. For the further analysis of curve progression, all included patients with at least one year of radiographic follow-up were analyzed.

Additionally, patients were included from a database of a third specialized 22q11.2DS center, where patients with scoliosis frequently receive an MRI of the spine for indications such as pain, fast progression, or pre-operative screening. Patients with congenital spinal anomalies or with only post-operative MRIs were excluded. These MRIs were analyzed for intraspinal anomalies.

### 2.2. Radiographic Analysis

**Figure 1:**
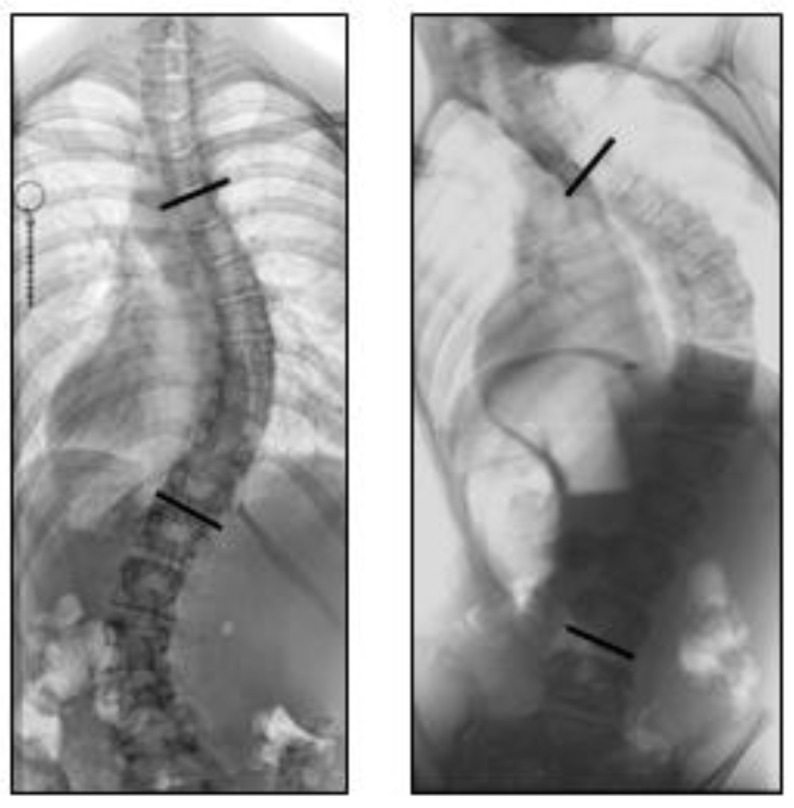
Two examples of different scoliosis curve types. On the left is an idiopathic-like curve, which is S-shaped with the apex of the major curve located at vertebral level T9 and a curve length of 8 vertebrae. On the right is a non-idiopathic neuromuscular-like curve, which is C-shaped with a curve length of 12 vertebrae, and a lower-end vertebra located at level L4.

One trained and experienced observer (JH), blinded for all other clinical parameters, analyzed all radiographs in chronological order. First, the Cobb angle was measured of all scoliotic curve(s), and the location of the major curve (i.e., the largest) was noted as either thoracic (apex at T2 – disc T11/T12), thoracolumbar (apex at T12 – L1), or lumbar (apex at disc L1/L2 – L4), according to the Scoliosis Research Society guidelines [21]. Next, curve morphology was determined based on the first available radiograph of each patient, according to the criteria determined by Abul-Kasim et al. in 2010 [23]. Curves were classified as non-idiopathic if three conditions were met: (1) the Cobb-to-Cobb segment exceeded eight vertebrae, (2) the curve was C-shaped, and (3) the curve included the lowest or second-lowest lumbar vertebra (Figure 1). The findings in the 22q11.2DS patients in this study were compared to reference observations in idiopathic and non-idiopathic scoliosis patients in the general population [23].

Furthermore, the progression of scoliosis curve severity was analyzed by measuring the Cobb angle on the first and last radiograph available for each patient with at least one year of follow-up. A progressive curve was defined as at least a 5° increase over the follow-up period [24]. Additionally, if a patient had received brace treatment or surgery, the curve was considered progressive. Of all 22q11.2DS patients with progressive scoliosis, the curve progression rate in degrees of Cobb angle per year was calculated and compared to reference values of idiopathic scoliosis in the general population, as described in a meta-analysis by Di Felice et al. in 2018 [24].

### 2.3. MRI Analysis

Of the spinal MRIs made of 22q11.2DS patients with scoliosis, all reports were screened for the presence of intraspinal anomalies and annotated as described by the clinically involved radiologist at the time of investigation. The rate of intraspinal anomalies in 22q11.2DS patients with scoliosis was compared to that reported in idiopathic scoliosis in the general population, as described in a meta-analysis by Heemskerk et al. in 2018 [25].

### 2.4. Statistical Analysis

The age at diagnosis, sex, curve progression (< or >5°), and presence of spinal anomalies in the 22q11.2DS patients in this study were compared to data on idiopathic scoliosis in the general population from literature. Normality of distribution was tested with Q–Q plots, the means ± standard deviations were calculated for normally distributed variables, and medians and interquartile ranges (IQR) were calculated for not normally distributed variables. Since data were compiled from multiple cohorts with different criteria, no comparative statistics were performed to produce irrelevant *p*-values. The descriptive statistical analyses were performed with SPSS 25.0 for Windows (IBM, Armonk, NY, USA).

## 3. Results

### 3.1. Study Population

**Figure 2:**
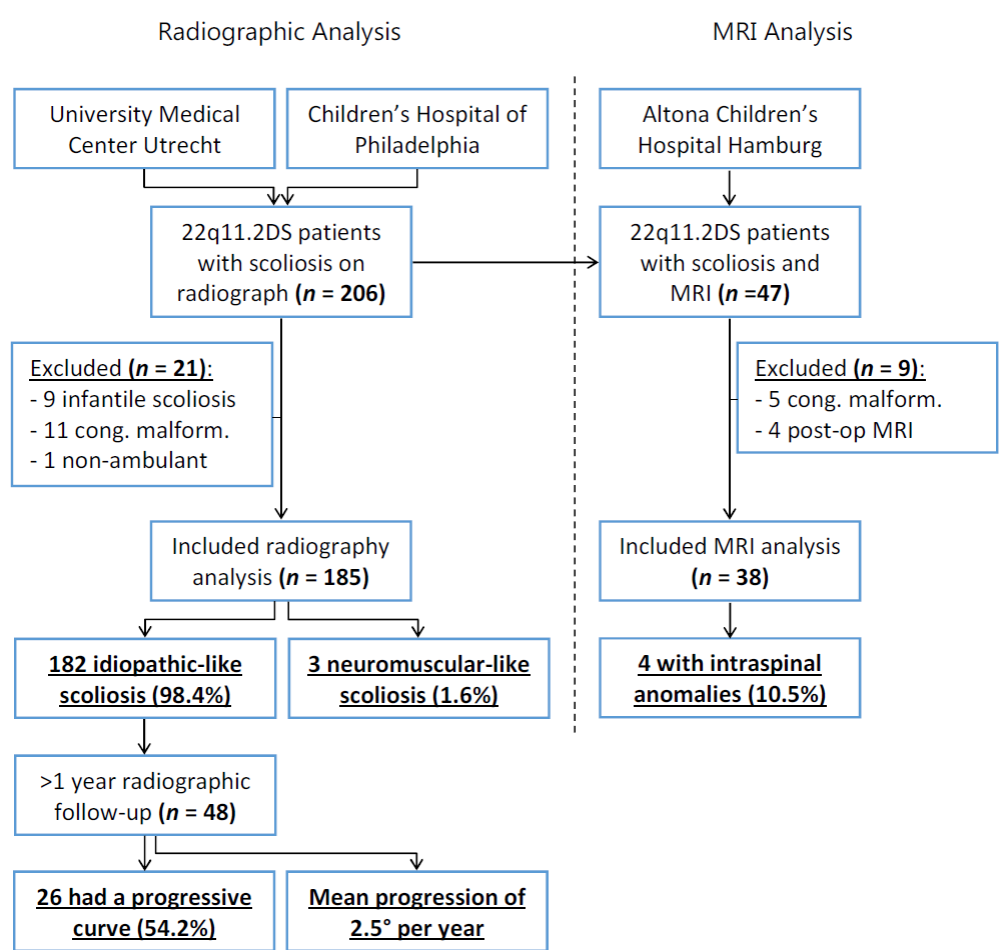
Flowchart shows the inclusion and exclusion of patients in the current study. Furthermore, the results of both the radiographic and MRI analysis are displayed.

From two databases, 206 patients with 22q11.2DS, scoliosis, and a full spine radiograph were retrieved; after 21 exclusions, 185 patients were included for radiographic analysis (Figure 2). From these 185 patients, a further 48 had at least one year of radiographic follow-up and were included for analysis of their scoliosis curve progression. Finally, for the MRI analysis, after nine exclusions, 38 patients were included for analysis of the rate of intraspinal anomalies (Figure 2).

**Figure 3:**
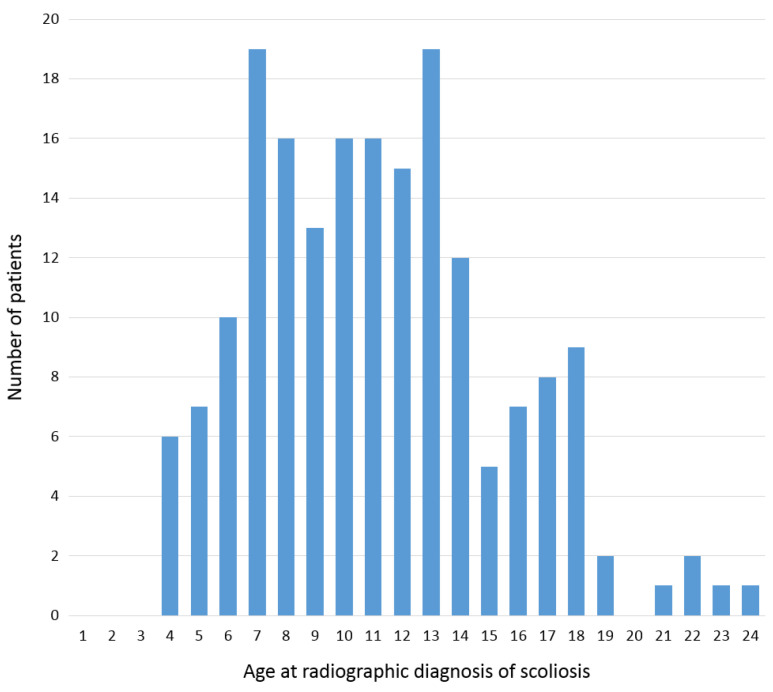
The age distribution at the moment of initial radiographic diagnosis of scoliosis. In the 185 included 22q11.2 deletion syndrome patients in this study, the mean age at diagnosis was 11.6 ± 4.2.

The mean age at diagnosis of scoliosis in 22q11.2DS patients was 11.6 ± 4.2, and 92 (49.7%) were female (Figure 3). In literature, the mean age at idiopathic scoliosis diagnosis in the general population varies due to different screening and diagnosis protocols but is reported 9.5–13.6 [26,27,28]. Furthermore, the ratio of females to males in idiopathic scoliosis is reported as 1.44 to 1, corresponding to 59% females [26].

### 3.2. Radiographic Analysis

Of the 185 patients with 22q11.2DS and scoliosis, the median Cobb angle was 16° (IQR: 13–25°). A total of 182 patients (98.4%) had an idiopathic-like curve based on Abul-Kasim’s criteria (Table 1) [23]. The other three patients (1.6%) fitted the criteria for neuromuscular-like scoliosis. Remarkably, the proportion of S-shaped curves was 69%, much higher than the 18% reported earlier for idiopathic scoliosis (Table 1) [23]. Of the 48 patients with at least one year of radiographic follow-up, the mean age at scoliosis diagnosis was 9.8 ± 2.7, and the median follow-up was 3.4 years (IQR: 2.3–5.1). There was a curve progression of at least 5° in 26 patients (54.2%), at an average progression rate of 2.5° per year, ranging from 1.4° to 5.0°. This is very comparable to idiopathic scoliosis, which has a reported proportion of 49% and a similar rate of 2.2–9.6° per year in the general population [24].

### 3.3. MRI Analysis

Out of 38 scoliosis patients with 22q11.2DS and an available MRI of the complete spine, four patients (10.5%) had a total of five intraspinal anomalies. The different anomalies were one tonsillar herniation, one extradural cyst, one intraspinal lipoma, and two vertebral body abnormalities. This is comparable to a prevalence of 11.4% in idiopathic scoliosis in the general population (Table 2) [25].

## 4. Discussion

The problem with the current etiology research on idiopathic scoliosis is that only established cases can be studied, and prospective research before the onset of the deformity is unfeasible in the general population [1,3]. The solution is the use of a ‘model’; however, currently, for idiopathic scoliosis, only ‘models’ of genetically or anatomically modified small animals exist [7,9,10,11,12]. This study investigated the relevance of a human ‘model’ for idiopathic scoliosis by using a subset of the population with a high risk for the disease. Children with 22q11.2DS have a 20-fold increased prevalence of scoliosis at around 50% and are usually prospectively followed from birth [14,17,18]. The purpose of this multicenter study was to analyze the morphology, dynamic behavior, and presence of intraspinal anomalies in scoliosis patients from 22q11.2DS cohorts in comparison to idiopathic scoliosis.

Over two hundred patients with 22q11.2DS and scoliosis were compared to thousands of patients with idiopathic scoliosis from the recent literature. The mean age at diagnosis of scoliosis in patients with 22q11.2DS was 11.6, and 49.7% were females. In idiopathic scoliosis, this is reported as 9.5–13.6 years old and 59% females [26,27,28]. This broad range of reported age at diagnosis is caused by the many different scoliosis screening protocols in different countries. However, if scoliosis in 22q11.2DS onsets at the same age as idiopathic scoliosis, the mean age at diagnosis in patients with 22q11.2DS is likely to be lower since spinal radiography is part of the standard follow-up, promoting early diagnosis. The vast majority of included patients with 22q11.2DS and scoliosis (98.4%) had a curve morphology that was consistent with predefined criteria for an idiopathic curve [23]. Additionally, the proportion of progressive curves (54.2%) and the rate of curve progression (2.5° per year) was similar to reports on idiopathic scoliosis, with 49% being progressive at a rate of 2.2–9.6° per year [24]. Finally, the prevalence of intraspinal anomalies in 22q11.2DS patients with scoliosis was 10.5%, which was similar to the 11.4% prevalence in idiopathic scoliosis in the general population [25].

There are multiple classification systems that describe the curve morphology pattern in scoliosis. The well-known King and Lenke classifications were created mainly for surgical planning by distinguishing stiffer/structural curves from non-structural curves rather than distinguishing between idiopathic and non-idiopathic scoliosis [29,30]. Abul-Kasim et al. showed that non-idiopathic curves display distinct morphologic characteristics on upright standing spinal radiographs, including the scoliosis shape, the curve length, and the contribution of the lowest lumbar vertebrae to the curve (Figure 1). These characteristics were translated into three criteria to distinguish idiopathic from non-idiopathic curves, which were used in this study of 22q11.2DS scoliosis [23]. While these three criteria are all assessed from anterior–posterior standing spinal radiographs, idiopathic scoliosis is a 3D deformation of the spine and trunk, including vertebral rotation and sagittal plane deformation. However, since lateral radiographs were not routinely made in all participating centers, these were not analyzed in this study. Furthermore, accurate assessment of the sagittal spinal profile is notoriously unreliable on lateral radiographs, especially in more severe curves with a larger Cobb angle, due to coupling of the spinal curvature in all three planes [31,32].

Besides the global curve morphology criteria for idiopathic scoliosis, the curve behavior over time was also reckoned as relevant. Although similar values for the proportion and rate of curve progression were observed in 22q11.2DS scoliosis in comparison to idiopathic scoliosis, this should be interpreted with caution since scoliosis progression is heavily influenced by age, sex, curve location, and curve magnitude, all parameters that were not normalized or matched in this study [24,33]. Additionally, in the 22q11.2DS cohort, there was a median follow-up difference between the progressive curves (4.4 years) and non-progressive curves (2.5 years); therefore, the number of progressive curves in 22q11.2DS might be underestimated by this study. Future studies, with, for instance, an age- and sex-matched design, could aim to confirm the similarities in curve behavior between 22q11.2DS and idiopathic scoliosis. The addition of an MRI-based analysis to this study was mainly to exclude intraspinal anomalies as an important cause of scoliosis in 22q11.2DS. Indeed, the intraspinal anomaly prevalence was comparable to idiopathic scoliosis [25].

Although this study demonstrated similarities in curve morphology and behavior between scoliosis in 22q11.2DS and idiopathic scoliosis, the validity of 22q11.2DS as a ‘model’ has obvious limitations. First, the absence of typical non-idiopathic features that were identified for neuromuscular scoliosis does not imply that the curve is similar to idiopathic scoliosis. On the contrary, more subtle differences could be observed, such as the distribution between thoracic, thoracolumbar, and lumbar curves, as well as the contribution of L4 to the curve. Second, in this study, patients with congenital spinal anomalies that induced congenital scoliosis were excluded. This was because in the general population, and most likely also in 22q11.2DS, the pathoetiology of congenital scoliosis varies greatly from the development of idiopathic scoliosis [22]. It is known that in 22q11.2DS, the rate of congenital spinal malformations, especially in the cervical spine, is higher than the general population; therefore, if 22q11.2DS scoliosis were to be used as a ‘model’ to study idiopathic scoliosis, the congenital curves should be excluded [34]. Third, 22q11.2DS is a multisystem syndrome with many phenotypes resulting, for example, in hypocalcemia and a lower bone mineral density in half of the patients [14,35]. Interestingly, a proportion of patients with idiopathic scoliosis in the general population have lower bone mineral density [36,37,38,39]. Irrespective of a 22q11.2 deletion, a lower bone mineral density might be an independent risk factor for idiopathic-like scoliosis. Additionally, congenital heart disease is prevalent in 22q11.2DS, and for many decades, congenital heart disease has been linked to scoliosis in the general population [40,41]. Recent observations in different cohorts demonstrate that the 22q11.2 deletion itself is a confounder in this relationship and that in both the general population and in 22q11.2DS, congenital heart disease itself is not a large scoliosis risk factor [42]. Finally, children with 22q11.2DS differ from the general population in frequent phenotypes such as slow maturation, short stature, and articular laxity, which could all influence scoliosis development, but their exact effects are currently unknown. Future studies using 22q11.2DS scoliosis as a model should aim to normalize these as much as possible, for instance, by determining the individual offset from maturity, i.e., the difference between chronological age and biological maturity [43].

For a scientific ‘model’ to be valid, the disease of the ‘model’ must sufficiently resemble the condition in the general population before it can be used to study etiological aspects of the disorder. Of course, any ‘model’ is at best an approximation of the true disease; this holds true for the often-used animal model as well. Scoliosis in 22q11.2DS does have differences from true idiopathic scoliosis in the general population, but this study demonstrated many important similarities in curve morphology and behavior. A future goal could be to utilize this ‘human model’ in prospective studies on idiopathic scoliosis etiology—for example, to study the spinal sagittal profile before scoliosis onset and its influence on scoliosis development [8,44]. Another option is to examine whole-genome sequencing in patients with scoliosis and 22q11.2DS, and those in the general population, as a clue to identifying the genomic etiology. Studying psychotic, ‘schizophrenia-like’ disorders in the 22q11.2DS population has yielded important information on idiopathic schizophrenia, a disorder that also seems exclusive to humans in the general population [13]. We propose to use the same approach in idiopathic scoliosis research.

## 5. Conclusions

To better understand idiopathic scoliosis etiology, prospective research on the pre-scoliotic spine is needed but is practically impossible in the general population. Animal models can help, but a validated human model would be superior. This study explored scoliosis in patients with 22q11.2DS as a possible human ‘model’ for idiopathic scoliosis. These patients have a 20-fold increased scoliosis risk and a curve morphology that resembles idiopathic scoliosis. Additionally, the curve dynamic behavior, in terms of prevalence and rate of curve progression and the prevalence of intraspinal anomalies, closely mimicked idiopathic scoliosis. This suggests that 22q11.2DS scoliosis may be a very relevant ‘model’ to prospectively study and help better understand certain aspects of idiopathic scoliosis etiology in the general population.

## Figures and Tables

**Table 1:** Curve characteristics of the 185 ambulant 22q11.2 deletion syndrome patients with scoliosis next to references values of the idiopathic and neuromuscular scoliosis. From Abul-Kasim et al. (2010).

	Current Study	Abul-Kasim et al. (2010) ^1^	
	22q11.2DS	Idiopathic Scoliosis	Neuro. Scoliosis
*n*	185	77	21
Major curve location			
- Thoracic	79 (43%)	52 (68%)	5 (24%)
- Thoracolumbar	54 (29%)	13 (17%)	12 (57%)
- Lumbar	52 (28%)	12 (16%)	4 (19%)
Median curve length in № vertebrae (IQR)	6 (5–7)	7 (7–8)	10 (9–11)
>8 vertebrae in curve	18 (10%)	3 (4%)	19 (90%)
Curve morphology			
- S-shape	127 (69%)	14 (18%)	0 (0%)
- C-shape	58 (31%)	63 (82%)	21 (100%)
Lower-end vertebra			
- L3+	114 (62%)	62 (81%)	5 (24%)
- L4	67 (36%)	15 (19%)	8 (38%)
- L5	4 (2%)	0 (0%)	8 (38%)
Fulfilled criteria for ^2^			
- Neuro. scoliosis	3 (2%)	0 (0%)	16 (76%)
- Idiopathic scoliosis	183 (98%)	77 (100%)	5 (24%)

^1^ Reprinted by Permission of SAGE Publications, Ltd. [23]. ^2^ The combination of a Cobb-to-Cobb curve length > 8 vertebrae, a C-shaped curve, and the location of the lower-end vertebrae at the lowest or second-lowest lumbar vertebrae is determined as a non-idiopathic (neuromuscular) curve pattern [23].

**Table 2:** Intraspinal anomalies in 38 patients with 22q11.22 deletion syndrome and scoliosis compared to idiopathic scoliosis in the general population, as described by Heemskerk et al. (2018).

	Current Study	Heemskerk et al. (2018) ^1^
	22q11.2DS	Idiopathic Scoliosis
*n*	38	8622
Spinal anomaly:		
-Isolated syrinx		318 (3.7%)
-Isolated Arnold-Chiari malf.		259 (3.0%)
-Arnold-Chiari malf. with syrinx		218 (2.5%)
-Tethered cord		49 (0.57%)
-Dural ectasia		33 (0.38%)
-Cerabral or intra/paraspinal tumors		22 (0.26%)
-Tonsilar herniation	1 (3%)	20 (0.23%)
-Diastematomyelia		19 (0.22%)
-Abnormal position of conus		10 (0.12%)
-Extra- or intradural cysts	1 (3%)	6 (0.07%)
-Intraspinal lipoma	1 (3%)	5 (0.06%)
-Discopathy		4 (0.05%)
-Hydrocephalus		3 (0.03%)
-Vertebral body abnormality	2 (5%) ^2^	3 (0.03%)^3^
-Hydromyelia		2 (0.02%)
-Craniocervical junctional narrowing		1 (0.01%)
-Cerebellar angioma		1 (0.01%)
-Dandy-Walker syndrome		1 (0.01%)
-Arteriovenous fistula		1 (0.01%)
-Not specified		88 (1%)

^1^ Compiled from the systematic review and meta-analysis as performed by Heemskerk et al. [25]. ^2^ Patient 1: Mild osteophytic ridging at T10-T11 and T11-T12 with Schmorl’s nodes and decreased disc height at these levels. Patient 2: Vertical cleft in the midline of the T7 vertebral body. ^3^ Hemangioma or lipoma in vertebral body.

## Data Availability

The data presented in this study are available on request from the corresponding author. The data are not publicly available due to local ethical guidelines.
